# Differences in static postural control between top level male volleyball players and non-athletes

**DOI:** 10.1038/s41598-020-76390-x

**Published:** 2020-11-09

**Authors:** Dorota Borzucka, Krzysztof Kręcisz, Zbigniew Rektor, Michał Kuczyński

**Affiliations:** 1 Faculty of Physical Education and Physiotherapy, Opole University of Technology, ul. Prószkowska 76, 45-758 Opole, Poland; 2 Faculty of Physiotherapy, University School of Physical Education in Wroclaw, Al. I.J. Paderewskiego 35, 51-612 Wrocław, Poland

**Keywords:** Motor control, Sensorimotor processing, Attention

## Abstract

It is argued that elite athletes often demonstrate superior body balance. Despite the apparent significance of perfect balance ability in volleyball, little is known about the specific nature of postural control adjustments among first-rate volleyball competitors. This study compared postural performance and strategies in quiet stance between world vice-champions and young, healthy, physically active male subjects. The center-of-pressure (COP) signals recorded on a force plate were used to compute several measures of sway. In both axes of movement, athletes had lower COP range, but not its standard deviation and higher COP speed and frequency than controls. These findings indicate that postural regulation in athletes was more precise and less vulnerable to external disturbances which support optimal timing and precision of actions. Postural strategies in athletes standing quietly were similar to those exhibited by non-athletes performing dual tasks. It demonstrates a significant effect of sport practice on changes in postural control. In anterior–posterior axis, athletes displayed a much higher COP fractal dimension and surprisingly lower COP–COG frequency than controls. This accounts for their high capacity to use diversified postural strategies to maintain postural stability and significantly reduced the contribution of proprioception to save this function for carrying out more challenging posture-motor tasks.

## Introduction

Postural control is a complex motor skill derived from the interaction of multiple sensorimotor processes^1^ and combines regulation of stability and orientation to environment. It is important for most other motor activities, which are performed concurrently because people usually stand in order to accomplish a goal-directed task^2^. However, complex motor activities whose execution is based on the situational context and fast decision making, raise the demand for attentional resources and may cause very specific changes in postural control. Most frequently, such situations take place in sports that require a stable, robust control of balance as well as an adequate timing and precision of the concurrent motor action^3^. To optimally respond to such challenges, postural control should be highly flexible, affording a large spectrum of postural strategies to cope with unpredictable perturbations^4^. Also, it would require increased automaticity to support relocating attentional resources to the concurrent motor task. The achievement of such a superior postural control may result only from long-term specific training^5^. Therefore, the investigation of postural control of athletes in sports that put high demands on body stability can elucidate some postural mechanisms and strategies which are still poorly understood.

Not surprisingly, the most stimulating results were derived from top-level athletes because postural ability often reflects athletic skill level^6^. The research into postural stability of elite professional athletes is scarce and relatively new except the investigation of postural stability in shooters^7,8^ who consistently showed reduced sway velocity. Significantly decreased sway amplitude has also been found in elite soccer players^9^, gymnasts^10,11^, golfers^12^, and hockey players^13^. These results are encouraging for further research and show that intensive specialized training may lead to superior postural performance.

Apart from soccer, surprisingly little attention has been devoted to the significance of postural control in team sports and in volleyball in particular^14,15^. The reason for this oversight may be the apparent impression that body balance does not significantly contribute to progress in this game. Indeed, for a casual observer, many points scored or defended in volleyball are the results of airborne actions. However, an equally important contribution to the final score is being made by less spectacular actions including passing, setting, and digging that require perfect anticipation and timing, which, in turn, rely on stable, optimally aligned body position^16^. In all volleyball actions during which the player has contact with the floor, maintaining postural stability is crucial. The high effectiveness of these actions is determined by the player’s ability to control his or her postural sway. High robustness to any disturbances of body balance is necessary as actions with the ball without adequate postural stability are far less accurate. This is because the central nervous system aims first at restoring the body vertical which may interfere with the optimal execution of suprapostural task.

Volleyball is highly challenging to players as they must hit or pass the ball while being in highly dynamic and often unstable postures.

Thus, due to the inevitable trade-off between stability and maneuverability^17,18^, exceptional postural control is a prerequisite for volleyball players. Most of the actions on the court have a ‘destabilization–recovery of balance’ sequence that boost demands not only on spatial but also on temporal balance abilities^19^. The static stability is continuously challenged by the demand for an optimal body position prior to actions with the ball. Thus, the postural sway variability should be limited to ensure precision and efficiency in action, but should also provide the CNS with adequate exploratory capabilities to cope with changing situation on the court. Somewhat different balance skills are crucial in ensuring good stability in approaching and landing for spiking or blocking jumps and also in controlling body posture in aerial phases of spiking^20^. The need to satisfy the latter apparently conflicting demands on postural stability may shape a very unique static postural control in volleyball players. The recognition of such explicit postural behavior in these athletes may help understand its significance in enhancing performance on court and shed light on possible ways to promote this specific postural control. It might also be used as an additional training goal and an index of personal progress in volleyball.

The purpose of this study was to compare static postural control in elite Polish male volleyball players with a control group of young males who were not involved in any systematic sports activity. We hypothesized that the athletes would have better postural performance and a higher level of sway complexity, which is characteristic of a larger repertoire of postural strategies. We expected that such an improvement in balance could not be achieved without some changes in postural strategies. Thus, we also hypothesized a higher rate of exploratory function in athletes that would manifest in the increased frequency of postural corrections.

## Methods

### Participants

Thirty-one players of the Poland national men's volleyball team (age 24.3 ± 3.3 yr; height 197.4 ± 7.5 cm; weight 91.6 ± 8.9 kg) were examined. The balance assessment was carried out at the team camp a few months before the World Cup, which took place in November and December 2006 in Japan. It was a tactical camp. The intensity and volume were small. The volleyball players trained twice a day for 1.5 h. The tests were performed before the first training session, after a night of rest, without warming up, without any previous tests, no one had participated in such tests before. Selected players from this group won a silver medal. The research was conducted with the consent of the Polish Volleyball Association, training staff and players. The volleyball players did not have any postural impairments. In the last 1.5 years, none had a sprained ankle. The control group consisted of 31 young men (age 22.9 ± 1.3; body height 180.9 ± 6.5 cm; body weight 76.9 ± 9.9 kg). These were students of the Faculty of Physical Education and Physiotherapy, who did not undertake systematic physical activity. All of the students described their health condition as very good and agreed to participate in the study. All subjects gave an informed written consent and the study was approved by the Bioethics Committee of Opole Chamber of Physicians in Opole. Students responded to a short questionnaire and those whose exercise physical activity was less than three times a week and less than 150 min a week were included to the control group. The students never had any ankle injuries and no postural defects. The tests were performed without warming up, without any previous tests, no one had participated in such tests before.

### Measurement system

Ground reaction forces were acquired with a custom-made force plate with strain-gauge full bridge transducers which were amplified and fed into an IBM PC through a 12-bit A/D converter. The signals were digitally recorded with a custom-made software at sampling rate of 20 Hz.

### Study design

The study protocol was the same as at^14^. Participants were measured on a force plate with eyes open, and their COP signals were recorded for the 20 s in the medial–lateral (ML) and anterior–posterior (AP) planes. The participants were requested to stand barefoot, with their arms at sides. All methods were performed in accordance with the relevant guidelines and regulations of the Bioethics Committee of Opole Chamber of Physicians in Opole.

### Parameters

On the basis of the recorded COP signals, several parameters describing different properties of the postural control system were computed and compared between both groups. This included the measures of the COP variability^21^: standard deviation (SD), range (RA), and mean velocity (MV), and the indices of postural performance and complexity: peak frequency (PF) of COP-COM (center of mass) correction signal, COP frequency (CF) based on normalized path COP length and fractal dimension (FD) which quantifies the degree to which COP time series fills the metric time–space. Specifically, the peak frequency was derived from parameters of the viscoelastic model of standing posture whose detailed definition is covered in Kuczyński^22^, whereas the COP frequency is defined as:

$$CF=\frac{MV}{2\cdot \pi \cdot SD}$$

and fractal dimension:

$$FD=\frac{\mathrm{log}10\left(400\right)}{\mathrm{log}10\left(400\cdot \frac{RA}{MV\cdot 20}\right)}$$

where 400 is the number of samples (20 s times 20 Hz).

### Statistics

Due to low p-values values of Shapiro–Wilk tests of normality, all dependent variables were subjected to Mann–Whitney U group independent tests in the anterior–posterior and medio-lateral plane separately. The effect size statistic for the Mann–Whitney test is *r* is the *Z* value from the test divided by the total number of observations.: small 0.1– < 0.3, medium 0.3– < 0.5, large ≥ 05. Statistical evidence of significance was set at *p* < 0.05. All tests were conducted with free and open software JAMOVI, Version 1.2 (retrieved from https://www.jamovi.org) and in R with rcompanion^23^.

## Results

The results of the experiment are presented in Table 1. Athletes had a significantly lower range of the COP, similar variability and higher mean velocity of postural oscillations than controls. The frequency of postural sway is increased, which was accompanied by a high fractal dimension in both planes.

**Table 1:** Group descriptives and Mann–Whitney U independent tests for groups comparison (N = 31).

	Group	Mean	SD	Median	Quartiles	W statistic	*p*	r effect size
**AP**								
SD (mm)	Students	4.62	2.22	4.09	2.86–5.12	411	0.333	0.12
	Athletes	3.98	1.34	3.94	3.10–4.55			
RA (mm)	Students	22.76	7.93	21.30	16.55–25–69	290	**0.007**	0.34
	Athletes	17.71	4.85	17.79	15.70–20.34			
MV (mm/s)	Students	6.65	1.94	6.40	5.22–7.54	292	**0.007**	− 0.34
	Athletes	7.63	1.51	7.65	6.78–8.14			
PF (Hz)	Students	0.59	0.12	0.55	0.53–0.67	229.5	** < .001**	0.45
	Athletes	0.47	0.12	0.47	0.41–0.54			
CF (Hz)	Students	0.26	0.11	0.23	0.17–0.30	275	**0.003**	− 0.37
	Athletes	0.33	0.11	0.31	0.25–0.40			
FD (–)	Students	1.43	0.11	1.41	1.34–1.50	174	** < .001**	− 0.55
	Athletes	1.58	0.12	1.59	1.49–1.65			
**ML**								
SD (mm)	Students	3.05	0.91	2.90	2.35–3.49	438	0.549	0.08
	Athletes	2.94	1.11	2.78	2.10–3.59			
RA (mm)	Students	17.39	4.86	17.58	13.78–19.92	323	**0.026**	0.28
	Athletes	14.64	5.73	14.06	10.78–17.96			
MV (mm/s)	Students	5.65	1.57	5.38	4.78–6.58	331	**0.035**	− 0.27
	Athletes	6.69	1.87	6.19	5.29–8.02			
PF (Hz)	Students	0.66	0.14	0.66	0.60–0.74	452	0.695	− 0.05
	Athletes	0.69	0.17	0.68	0.59–0.74			
CF (Hz)	Students	0.31	0.10	0.30	0.24–0.39	290	**0.007**	− 0.34
	Athletes	0.39	0.11	0.37	0.31–0.46			
FD (–)	Students	1.46	0.11	1.45	1.39–1.51	158	** < .001**	− 0.58
	Athletes	1.61	0.11	1.56	1.54–1.67			

SD, standard deviation of COP; RA, a range of COP; MV, mean speed of COP; PF, peak frequency of COP-COM; CF, COP frequency; FD, fractal dimension; AP, anterior–posterior plane; ML, medial–lateral plane.

Overall, the Mann–Whitney *U* tests showed that there was statistical evidence of significance for the difference between groups for RA, MV, CF, and FD in both planes. For PF, only ML was affected by group. SD was not affected by group.

## Discussion

The purpose of this study was to compare postural control between elite male athletes specialized in volleyball (EVP) and their healthy counterparts who were not involved in any systematic sports training. As long as the traditional sway measures are concerned, we expected better postural steadiness in athletes. This applies to lower values of the COP amplitude and speed that reflect better postural performance with effortless and proficient postural control. We also expected a higher frequency of the COP–COM signal as a means supporting the steadiness of stance. Finally, we hypothesized higher COP fractality in athletes because it is recognized as evidence of greater adaptability and/or robustness to environmental demands.

Three findings are of particular interest showing unique postural control in athletes. They are only partly consistent with the hypothesized results. First, the EVP had lower COP range, but not its standard deviation, and higher COP speed and frequency in both axes of movement.

Second, contrary to our hypothesis, they displayed a lower frequency of the controlling signal (COP–COM) in AP plane. And third, they had a much higher COP fractal dimension.

It is rather unusual in stabilographic research to find significant differences in the ranges of the COP between two investigated groups and fail to find similar differences in the respective standard deviations. Both measures are always strongly correlated, as was also the case in this study (r = 0.91–0.92). With such high correlation coefficients, one could argue that both measures represent the same properties of the COP signal, namely its variability. Yet, considering r^2^ for the latter values, there is still about 20% for some additional factor that affected the two measures differently in both groups of subjects. From those two measures, the standard deviation rather than the range is considered as a better indicator of signal variability because its value is averaged by the number of samples. On the other hand, the range can display an unexpectedly high value, if a random fluctuation occurs during the process of computerized stability assessment. Thus, one can deduce that having generally similar variability of the COP to that of controls, the EVP are much less vulnerable to accidental fluctuations and/or disturbances to posture. Such a robust regulation of standing supports optimal timing and precision of actions. This conclusion is consistent with other authors^3,9,24^ in that balance performance is specific to the conditions and situations imposed by sport-specific activities. Along similar lines, Gautier et al.^25^ proposed that gymnasts developed a unique ability to adapt their postural control more rapidly to the perceptive transition. Further, they claimed that the specific body sway dynamics in gymnasts promoted the remarkable steadiness of their heads. As the perceptive transition is more demanding on a volleyball court than in a stable environment of gymnasts, the EVP might have made even stronger postural adaptations. For the sake of methodology, these results imply that unusual patterns in data, like in this study, may turn out to be as meaningful as significant differences between subjects.

The decreased COP range, a definitely positive aspect of postural control, presented in elite athletes, did not occur alone but was associated with increased COP frequency and speed. This observation is rather counterintuitive because increased speed and frequency of sway are most often caused by the increased difficulty in maintaining postural tasks^22^. It simply does not seem possible that performing an easy standing task is more arduous for highly trained athletes than controls. Thus, it is mandatory to offer another explanation which assumes different control modes in the two groups. The evidence to support this idea comes partly from what was said above regarding more robust regulation of posture in elite athletes and their particular resilience to perturbations. On the other hand, similar results were found in changes in postural dynamics with increasing height threat^26–28^. Subjects standing on an elevated force plate and exposed to a threat of falling substantially decreased their COP variability with the simultaneous increase in COP power spectrum. Although the physiological and psychological reasons for these similar mechanistic outcomes in elite athletes and subjects standing at heights are entirely different, they have a comparable behavioral goal. This goal is to minimize sway amplitude in order to promote subjective safety at heights and an optimal starting position in the volleyball court. However, low sway amplitude impairs the exploratory function of sway, and other means are necessary to support this activity. A good candidate is higher COP speed, which, by providing faster information regarding body position, makes up the disadvantage of the restricted exploration area.

A pertinent question arises about what factor increased COP speed while keeping the COP amplitude unchanged or even suppressed. The obvious answer provided by simple arithmetic is frequency^29^. However, typical COP signals display the combination of many components with different amplitudes and frequencies. A low-amplitude and high frequency component may significantly affect the sway speed, barely contributing to the measures of frequency^14^. On the other hand, a dominant component with relatively steady frequency would affect sway speed and frequency to the same extent. Such a dominant component is mechanically attributed to increased leg stiffness following co-contraction and is commonly used to explain changes in postural control with increasing height threat^28,30^. In contrast, it is useless in understanding postural strategy in a normal quiet stance of elite athletes, who demonstrated in the present study much lower COP–COM frequency than controls. The latter measure accounts for low ankle stiffness^31^ in elite athletes. It is consistent with Kim et al.^13^ who reported that muscle synergies in elite female ice hockey players in response to unexpected external perturbation exhibited low co-contraction between ankle agonists and antagonists. It also concurs with other findings that highly developed motor strategies are associated with low agonists-antagonists co-activation in various voluntary movements^32^. Taken together, elite athletes applied a different strategy to increase their COP speed, most likely based on specific changes in the temporal structure of their COP.

Further support for the latter argument is provided by authors investigating the effect of dual tasking and direction of attentional focus on postural control. Richer et al.^33^ reported that the external focus of attention reduced postural sway in young adults. An additional cognitive task led to an even greater reduction with an associated increase in COP speed and frequency. Interestingly, no effect of these conditions was found on muscle activity at the ankles, which was interpreted by Richer et al.^33^ as a manifestation of increased automaticity. It suggests a negligible role of stiffening strategy and closely corresponds with the results of elite volleyball players in this study. It also concurs with Kuczyński et al.^34^ who found lower COP variability and higher COP frequency, speed, and entropy, in elite competitive dancers. Using the entropy measure, they concluded that additional mental task promotes automaticity of postural control as does professional dancing compared to the sedentary controls. Comparable postural benefits of dual tasking were confirmed by other authors^35–37^.

In sum, there is strong evidence that combining postural with ancillary tasks, like a cognitive task, augmented sensory feedback, or an external focus of attention, improves postural performance in young adults. Remarkably, the EVP manifested the same beneficial pattern of postural sway measures in quiet stance yet, in the absence of any additional tasks. This suggests a unique postural control in athletes that capitalized on extensive training on the court where similar tasks are ubiquitous and consequently fine-tune postural strategies. The optimal control of EVP in a bipedal stance while waiting for the ball must take into account at least three sports skills. They incorporate the ability to judge the ball direction and speed, the readiness for transition from balance to action, and the anticipation of variable tactics. The balance-related prerequisites include benefitting from an external focus of attention, minimizing the deviation of body axis from the desired direction, and increasing automaticity of balance to afford more attentional resources for cognitive assignments.

The significant effect of volleyball-specific activities on the development of distinctive postural control in EVP is unmistakably reflected by a much lower frequency of the COP–COM signal as compared to controls. This result is in contrast to Kuczyński et al.^14^ who reported higher values of the same frequency in the second league volleyball players than in the non-training group. As both results were obtained from the same formula^22^ it is possible and creditable^38^ to make a direct comparison. The respective Hedges’ g as a measure of effect size produces the value around 1.35, which is indeed a very large difference that may account for paramount changes in postural strategies between low-level and elite volleyball players. The only plausible explanation that emerges from this comparison is a significant breakthrough in balance control abilities between the consecutive levels of sport expertise in volleyball. At the non-expert level, the increased sway frequency is the simplest measure promoting higher velocity that supports the more effective exploratory function of sway. However, the high rate of this exploration handicaps proprioceptive function in lower legs leading to its saturation^9^. Hence, sub-elite athletes become unable to compensate for more difficult postural tasks^4^. To successively cope at the highest level of sport performance, the elite volleyball players have to resort to less fatiguing and more adequate postural control strategies.

Finally, the large difference between EVP and controls in the COP complexity measure, the fractal dimension, may help resolve some ambiguities that were raised earlier. Higher sway complexity is considered an advantageous characteristic in the control of postural stability that is mainly reflected by the improved overall organization of postural control with better use of available sensory inputs^39^. These changes promote better adaptability to new postural challenges, demonstrating the capacity to use diversified postural strategies to maintain postural stability^40^.

In expert athletes, a greater contribution of vestibular information would reduce the contribution of proprioception, especially as part of easy and simple postural tasks. In this case, the proprioceptive function would be saved and would offer supplementary resources for carrying out more challenging postural tasks. Hence, expert athletes would have additional abilities to cope with destabilizing motor tasks^9^.

High sway complexity has been shown in ballet dancers and interpreted as a rearrangement of sensory integration and adaptation to extreme motor demands^41^. Also, increased fractality was reported as a result of balance training that was focused on the improvement of sensory reweighting^40^. It is plausible to assume that elite VP, who were frequently exposed to extreme motor challenges specific for this sport and often had to optimally share their sensory resources between posture and game, have developed unique postural control characterized by the high complexity of sway. The results of this study confirm the very specific characteristics of postural control in EVP. They must have evolved from the interaction of sports excellence, superior motor skills and relevant experience on the court. Superior body balance has never been considered as an ultimate goal for improvement simply due to the lack of definition of this superiority. For elite athletes, there are not prudent and invariable criteria or benchmarks for comparison. They would always vary apace with the varying challenges of specific sports. The results of this study appear to lend support to this premise.

**Figure 1:**
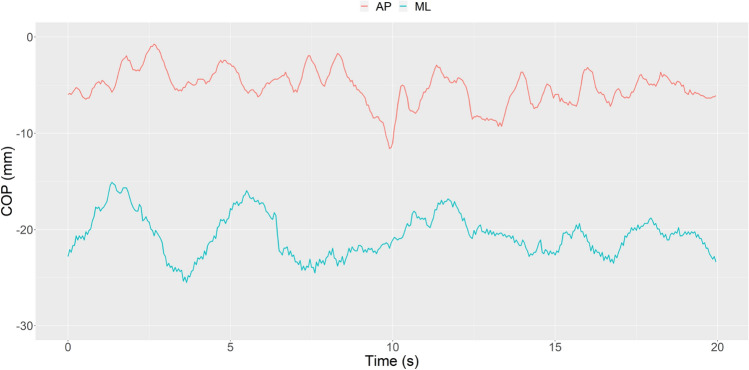
Example of stabilogram of center of pressure (COP) displacement during standing with eyes open. ML denotes medial–lateral and AP anterior–posterior plane.

Our study has some limitations. The sample size (N = 31 for both groups) is sensitive enough to detect r effect size 0.3 or larger only, with 80% power and 5% significance level. It is a medium effect size and our results must be interpreted accordingly. Small differences cannot be detected with that study design, so it poses some problems (e.g., sampling error as a source of bias etc.). Somewhat surprising results of our study may be attributed to this level of sensitivity. However, it is important to bear in mind the unique nature of our subject sample. Recruiting more top-ranking volleyball players is very hard. Another factor that limits our study is conducting only a quiet standing task with eyes open. Future studies comparing top-level players versus controls on several other standing tasks will be further informative to uncover other aspects of top-ranking volleyball player's postural control (Fig. 1).

## Data Availability

The datasets are available from the corresponding author on a reasonable request.
